# Vertical Levels of the Occipital Artery Origin

**DOI:** 10.3390/medicina59020317

**Published:** 2023-02-08

**Authors:** Cătălin Constantin Dumitru, Sorin Hostiuc, Alexandra Diana Vrapciu, Mugurel Constantin Rusu

**Affiliations:** 1 Division of Anatomy, Faculty of Stomatology, “Carol Davila” University of Medicine and Pharmacy, 050474 Bucharest, Romania; 2 Division of Legal Medicine and Bioethics, Faculty of Stomatology, “Carol Davila” University of Medicine and Pharmacy, 050474 Bucharest, Romania; 3 University Emergency Hospital Bucharest, 050098 Bucharest, Romania

**Keywords:** carotid artery, computed tomography, hyoid bone, gonial angle

## Abstract

*Background and Objectives.* The occipital artery (OA) is a posterior branch of the external carotid artery (ECA). The origin of the OA is commonly referred to a single landmark. We hypothesized that the origin of the OA could be variable as referred to the hyoid bone and the gonial angle. We thus aimed at patterning the vertical topographic possibilities of the OA origin. *Materials and Methods.* One hundred archived computed tomography angiograms were randomly selected, inclusion and exclusion criteria were applied, and 90 files were kept (53 males, 37 females). The cases were documented bilaterally for different levels of origin of the OA origin: type 1—infrahyoid; type 2—hyoid; 3—infragonial; 4—gonial; 5—supragonial; 6—origin from the internal carotid artery (ICA). *Results.* The incidence of unilateral types in the 180 OAs was: type 1—1.11%, type 2—5.56%, type 3—40.56%, type 4—28.33%, type 5—23.33% and type 6, ICA origin of the OA—1.11%. There was found a significant association between the location of the left and right origins of the OAs (Pearson Chi2 = 59.18, *p* < 0.001), which suggests the presence of a strong symmetry of the origins. Bilateral symmetry of the vertical types of the OA origin was observed in 56.67% of cases; in 43.33% there was bilateral asymmetry. *Conclusions.* The ICA origin of the OA is an extremely rare variant. For surgical planning or prior to endovascular approaches the topography of the OA origin should be carefully documented, as it may be located from an infrahyoid to a supragonial level.

## 1. Introduction

The upper neck vascular anatomy is needed for all medical practitioners performing skull base surgery, neurosurgery, vascular surgery, or orthopedic surgery [1]. Interventional radiologists, neurosurgeons, vascular, craniofacial, and neck surgeons must be aware of the anatomy of the external carotid artery (ECA) to perform an adequate interpretation of radiological images, for planning safe surgical approaches, and to execute surgeries such as carotid endarterectomies and carotid artery stenting [2].

Commonly, the ECA gives off the superior thyroid, lingual (LA), facial (FA), ascending pharyngeal (APA), occipital (OA), posterior auricular, maxillary and superficial temporal arteries. The OA may be a branch from the internal carotid artery (ICA), the thyrocervical trunk, the inferior thyroid artery, the vertebral artery, or the ascending cervical artery, according to Power (1850) [3] quoted in Bergman’s *Encyclopedia of Human Anatomic Variation* [4].

Nathan and Levy (1982) considered as a fixed landmark the origin of the OA from the posterior surface of the ECA opposite the origin of the facial artery, dissected 40 specimens and described three main types of OA—hypoglossal nerve relations: type I—OA origin superior to the 12th nerve, type II—OA origin on the same level and deep to the 12th nerve, and type III—OA origin below the level at which the hypoglossal nerve crosses the ECA [5]. In the drawings these authors provided, the OA origin from the ECA is represented at the level of the lingual artery origin, or immediately beneath that level [5]. The authors developed a theory explaining the respective patterns, mostly based on the developmental movement of the hypoglossal nerve [5]. The possibility of a vertical “gliding” of the OA origin from the ECA was seemingly overlooked. We therefore hypothesized that in adults the OA origin from the ECA could “glide” vertically from the carotid triangle to the retromandibular fossa. It was consequently aimed at studying the bilateral vertical topography of the OA origin as referred to osseous landmarks.

## 2. Materials and Methods

One hundred archived computed tomography angiograms (CTAs) were randomly selected. Inclusion criteria were the age of the subjects (>18 years), adequate quality of the CTAs, and no pathologic processes distorting the anatomy of the cervical carotid arteries. Exclusion criteria were pathological processes distorting the arterial anatomy and degraded or incomplete computed tomography scans. After excluding ten cases with incomplete or inadequate details on the ECA branches, there were kept for the study on 90 files. These were scans of 53 males and 37 females.

All subjects gave their informed consent for inclusion before they participated in the study. The research was conducted following principles from the Code of Ethics of the World Medical Association (Declaration of Helsinki). The responsible authorities (affiliation 3) approved the study (approval no.10540/16.02.2022).

The CTAs were performed with a 32-slice scanner (Siemens Multislice Perspective Scanner), with a 0.6 mm collimation and a reconstruction of 0.75 mm thickness with 50% overlap for a multiplanar maximum intensity projection and three-dimensional volume rendering technique, as described previously [6,7]. The cases were documented using Horos for iOS (Horos Project) [8]. Evaluations of the presence and subtypes of FPCAs were independently performed by an experienced anatomist (author #4) and an experienced oral and maxillofacial surgeon (author #1). The positive results were identical and were validated by each author.

The cases were documented bilaterally for the different vertical topographic patterns of OA origin, as referred to the greater horn of the hyoid bone and gonion: type 1—infrahyoid level of origin; type 2—hyoid level of origin (tip of greater horn); 3—infragonial level of origin; 4—gonial level of origin (tip of mandible’s angle); 5—supragonial level of origin; 6—origin from the ICA.

For evaluation of bilateral symmetry of the OA’s origin we defined several types’ combinations: type A—bilaterally symmetrical origins (with subtypes numbered according to the vertical type, A1, A2 etc.); type B—bilaterally asymmetrical origins of the OAs. The type B subtypes were numbered according to the respective unilateral vertical types (e.g., B2/3).

To evaluate the number of cases of each variant we used frequencies and the sex distribution. To evaluate significant associations between qualitative variables we used the Pearson Chi2 test. We used IBM SPSS Statistics software for Mac (IBM, Chicago, IL, USA) to perform statistical analyses.

## 3. Results

**Figure 1:**
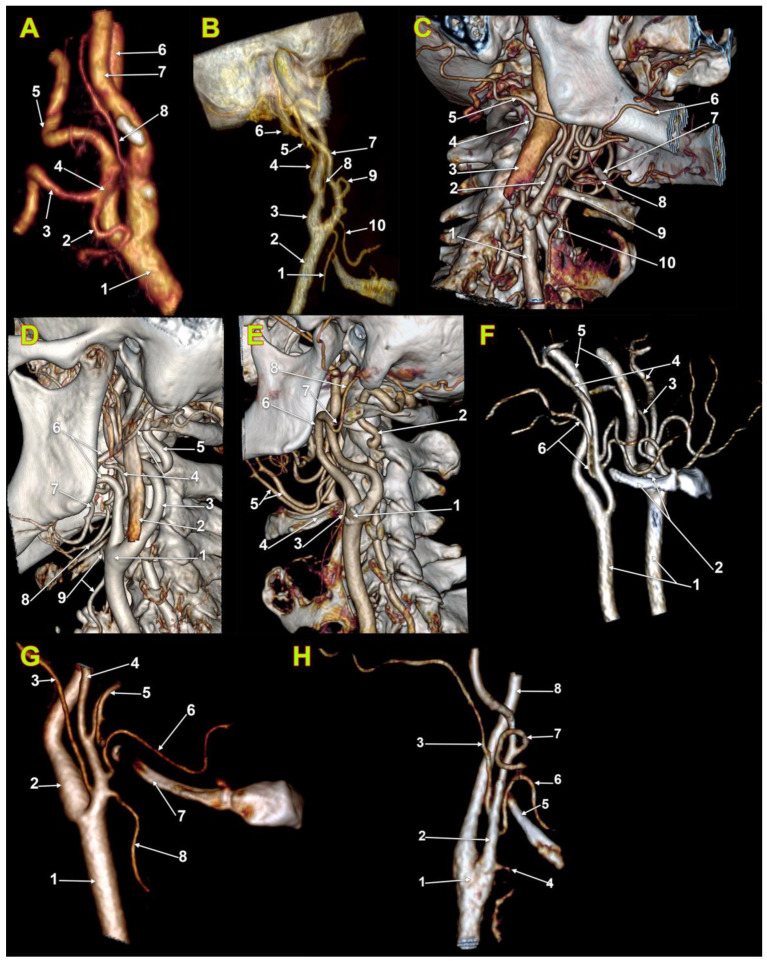
Computed tomography angiograms, three-dimensional volume renderings. (**A**). Origin of the occipital artery from the internal carotid artery. Right side. Postero-medial view. 1. common carotid artery; 2. lingual artery; 3. facial artery; 4. linguofacial trunk; 5. external carotid artery; 6. internal jugular vein; 7. internal carotid artery; 8. occipital artery. (**B**). Origin of occipital and ascending pharyngeal arteries from internal carotid artery. Right side. Infero-lateral view. 1. superior thyroid artery; 2. common carotid artery; 3. internal carotid artery; 4. occipital artery; 5. posterior auricular artery; 6. internal jugular vein; 7. internal carotid artery; 8. ascending pharyngeal artery; 9. facial artery; 10. lingual artery. (**C**). Infragonial (type 3) origin of the right occipital artery. Right side. Infero-lateral view. 1. common carotid artery; 2. external carotid artery; 3. internal jugular vein; 4. vertebral artery; 5. occipital artery; 6. facial artery; 7. contralateral ceratohyal bone; 8. lingual artery; 9. greater horn of hyoid bone; 10. superior thyroid artery. (**D**). Gonial (type 4) origin of the left occipital artery. Left side. Postero-infero-lateral view. 1. carotid bifurcation; 2. internal jugular vein; 3. internal carotid artery; 4. occipital artery; 5. vertebral artery; 6. external carotid artery; 7. facial artery; 8. lingual artery; 9. superior thyroid artery. (**E**). Origin at supragonial level (type 5) of the occipital artery. Left side. Infero-lateral view. 1. carotid bifurcation; 2. vertebral artery; 3. superior thyroid artery; 4. left greater horn of hyoid bone; 5. lingual arteries; 6. external carotid artery; 7. posterior auricular artery; 8. occipital artery. (**F**). Origin at the hyoid level (type 2) of the right occipital artery. Bilateral asymmetry of occipital artery origin. Right side. Antero-lateral view. 1. common carotid arteries; 2. greater horns of hyoid bone; 3. left occipital artery (suprahyoid origin); 4. external carotid arteries; 5. internal carotid arteries; 6. right occipital artery (hyoid origin). (**G**). Infrahyoid (type 1) origin of the occipital artery, at the same level with the origin of the superior thyroid artery. Right side. Antero-lateral view. 1. common carotid artery; 2. internal carotid artery; 3. occipital artery; 4. external carotid artery; 5. facial artery; 6. lingual artery; 7. greater hyoid horn; 8. superior thyroid artery. (**H**). Infrahyoid (type 1) origin of the occipital artery, above the level of the lingual artery origin. Right side. Antero-infero-lateral view. 1. carotid bifurcation; 2. external carotid artery; 3. occipital artery; 4. superior thyroid artery; 5. greater horn of the hyoid bone; 6. lingual artery; 7. facial artery; 8. internal carotid artery.

We identified in the overall batch the possibility of vertical variation in OA origin from infrahyoid to supragonial level (types 1–6) (Table 1 and Table 2). There were 180 determinations, bilaterally. Of these, in two male cases, we found unilaterally, on the right side, the OA origin from the ICA (2/180, 1.11%) (Figure 1A,B) In one of these two cases also the ascending pharyngeal artery left the ICA (Figure 1B). Type 3, OA origin between the hyoid and gonion (Figure 1C), was identified for 40.56% of OAs (73/180). Type 4, OA origin at the gonion (Figure 1D), was identified for 51/180 OAs (28.33%). Type 5, supragonial origin of OA (Figure 1E), was present for 42/180 OAs (23.33%). Type 2, hyoid level of origin (Figure 1F), was present for 10/180 OAs (5.56%). Type 1 (infrahyoid origin of OA) (Figure 1G,H) and type 6 (OA origin from ICA) (Figure 1A,B), respectively, were identified each for 2/180 OAs (1.11%).

There are no statistically significant associations between sex and the origin of the OA on the right side (Pearson Chi2 = 5.07, *p* = 0.407). There are no statistically significant associations between sex and the origin of the OA on the left side (Pearson Chi2 = 1.54, *p* = 0.671).

In males (n = 53), on the right side, we did not identify type 1—OA’s origin inferior to the level of the greater hyoid horn. Type 2, OA’s origin at the hyoid level, was present in 3.77% of cases, and type 3—OA’s origin superior to the greater hyoid horn but inferior to the gonion, was detected in most cases (39.62%). Type 4, gonial level of OA’s origin from the ECA, was found in 22.64%. Type 5, supragonial origin of OA from ECA, was found in 30.19% of cases. Type 6, OA’s origin from ICA, was present in 2/53 of the right side (3.77%) of the male group. On the left side in males (n = 53) we did not find types 1 (infrahyoid) and 6 (internal carotid origin of AO); type 2 was present in 7.55%, type 3 in 49.06%, type 4 in 26.42% and type 5 in 16.98% (Table 2).

On the right side, in women (n = 37) we did not identify the ICA origin of AO. Type 3 (infragonial) OA was present in 32.43%. Types 4 (gonial) and 5 (supragonial) were each identified in 29.73% of cases. Type 1, infrahyoid, was found in 5.41% and type 2, hyoid, in 2.7%. However, the two cases with type 1 origins of the OAs were different: in one case the OA originated the ECA at the level of the superior thyroid artery (Figure 1G), while in the other type 1 case the OA left the ECA distally to the LA (Figure 1H). On the left side, in females (n = 37), we did not find type 1 (infrahyoid) and type 6 (origin of OA from ICA). Type 3, infragonial, was present in 37.84% and type 4, gonial, also in 37.84%. Type 5, supragonial, was detected in 16.22%. Type 2, hyoid, had an incidence of 8.11% (Table 2).

There was found a significant association between the location of the left and right origins of the OAs (Pearson Chi2 = 59.18, *p* < 0.001), which suggests the presence of a strong symmetry of the OA’s origins. However, even if the results are statistically significant, there are numerous cases in which the origin is different on each side, being usually shifted by one vertical position (types Bx/x + 1).

**Figure 2:**
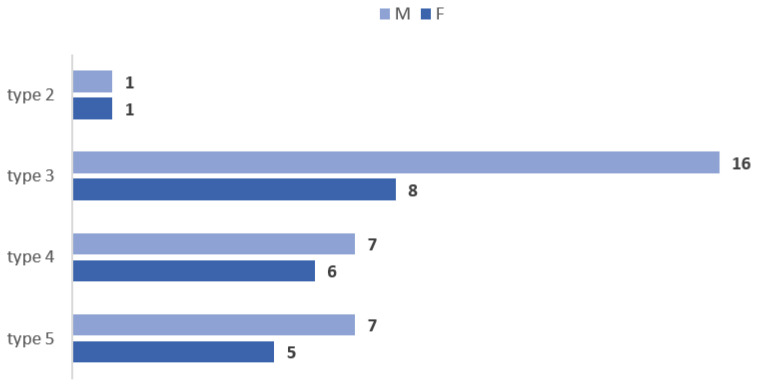
Sex distribution of bilaterally symmetrical types of vertical origin of the occipital artery. There was not found bilateral symmetry for types 1 and, respectively, 6. M: males; F: females.

Bilateral symmetry of the vertical topography patterns of the OA origin was observed in 51/90 (56.67%) of cases. In 43.33% of cases this variable showed bilateral asymmetry. Of the 51/90 cases with bilateral symmetry this was not detected for types 1 (infrahyoid type of the OA origin) and 6 (OA origin from ICA). In 2/51 of these cases type 2 showed bilateral symmetry (3.92%). In 24/51 cases (47.05%) there was bilateral symmetry for type 3, infragonial, of the OA. In 13/51 cases type 4 was bilaterally symmetrical (25.49%). In 12/51 (23.52%) we found bilateral symmetry of type 5, supragonial origin of OA. The sex distribution (M/F) of these four types with bilateral symmetry was: type 2—1/1, type 3—16/8, type 4—7/6, and type 5—7/5 (Figure 2).

There were 39/90 (43.33%) cases with bilateral asymmetry of the OA types. The subtypes (anatomical combinations) of cases with bilateral asymmetry identified were B1/2 (1/39, 2.56%), B1/5 (1/39, 2.56%), B2/3 (3/39, 7.69%), B2/4 (2/39, 5.12%), B3/4 (13/39, 33.33%), B3/5 (8/19, 20.51%), B3/6 (1/39, 2.56%), B4/5 (9/39, 23.07%) and B4/6 (1/39, 2.56%). In men we did not identify the combinations B1/2 and B1/5. In women we did not identify the combinations B2/4, B3/6 and B4/6.

In men we found 22 cases with bilateral asymmetry: type B3/5 in 7/22 cases (31.82%), type B3/4 in 5/22 cases (22.73%), type B4/5 in 4/22 cases (18.18%), types B2/3 and B2/4 in 2/22 cases (9.09%) each, and types B3/6 and B4/6 in 1/22 cases (4.55%) each.

In women we found 17 cases with bilateral asymmetry: type B3/4 in 8/17 cases (47.06%), type B4/5 in 5/17 cases (29.41%), and types B1/2, B1/5, B2/3 and B3/5 in 1/17 cases (5.88%) each.

## 4. Discussion

Regarding the vertical level of origin of the OA, *Gray’s Anatomy* only indicates that it originates in the neck from the ECA [9]. Rouvière and Delmas only state that the OA originates from the posterior aspect of the ECA, at the level of the FA or, more rarely, at the level of the LA [10]. In Sobotta’s atlas the origin of the OA was drawn halfway up the mandibular ramus [11]. Similarly, Pernkopf shows it in his atlas with retromandibular origin, thus higher than the origin of the FA [12]. In Grant’s atlas, the origin of the OA is depicted immediately posteroinferior to the tip of the greater horn of the hyoid bone [13]. Therefore, in different textbooks and anatomical atlases the description of the OA origin is not homogenous.

A number of other authors describe the origin of the OA associated with a single vertical level, or landmark, as follows. Newton and Young (1968) and Özgür et al. (2017) described that the OA normally branches from the ECA at the level of the FA [14,15]. Barral and Croibier (2011) described that the OA arises in the neck, anterior to the mastoid process, from the posterior aspect of the ECA and “branches off the carotid artery slightly above the FA” [16]. Various other authors have described the origin of OA at the gonion [17,18,19,20]. We found a gonial level of the OA origin in just 28.33% of sides.

Uchino and Saito (2020) indicate the origin of the OA from the proximal segment of the ECA [21]. Alvernia et al. (2006) describe the origin of OA “usually proximal to the origin of the FA” [22]. Kawashima et al. (2005) indicate the origin of the OA opposite the origin of the facial artery and close to the inferior margin of the posterior belly of the digastric muscle [23].

Different levels of origin of the OA, as referred to the hyoid bone and gonial angle, were assessed by the present study. These demonstrate that the OA does not have a constant vertical level of origin from the ECA, neither has it had a single landmark for a safe identification. This is in accordance with Acar et al. (2013), who observed that the OA “originated from almost every level of the ECA” [24]. The OA was divided by Seker et al. (2010) into three portions: ascending cervical; cervico-occipital; and ascending occipital [20]. A higher vertical level of the origin of the OA will shorten, or eliminate, the first, ascending cervical, portion of the AO, or determine kinks or coils of the OA.

Cobiella et al. (2021) studied 193 cadavers and observed the OA originating either from the ECA (94.7%), or from the carotid bifurcation (3.9%); the authors also observed that the OA could leave the ECA on any of its posterior, lateral, medial, or postero-lateral sides [25]. Different authors found the OA leaving the vertebral artery [26] or the vertebral artery arising from the OA [27]; we did not find such anatomical variants.

A dissection study identified the origin of the OA as referred to that of the FA and found different patterns: both arteries originating at the same level (75%), pharyngooccipital trunk originating proximal to the FA in 5%, origin of the OA proximal to the FA at the carotid bifurcation in 5%, and occipitoauricular trunk originating distally to the FA in 15% [28]. The respective authors did not follow bone landmarks correlating with variability of the ECA origin of the OA.

An anatomical study on a batch of 80 cadavers identified a number of patterns in the sequence of origin of ECA’s branches [29], as follows: the OA origin was proximal to LA’s origin in 10% of cases; at the level of the LA in 12.5%; distal to LA’s origin in 17.5%; at the level of the FA origin in 10%; distal to FA’s origin in 25%; proximal to a linguofacial trunk origin in 5%; at the same level as the origin of the linguofacial trunk in 1.3%; distal to the origin of the linguofacial trunk in 11.3%; proximal to the origin through a common orifice of the LA and FA in 1.3%; at the same level as the latter in 5%; and at the same level as the origin of the FA but in cases with thyrolingual trunks in 1.3% [29].

A recent meta-analysis on a set of 65 studies of OA documented that it starts from ECA as one of the first five branches; it originates as the fourth branch with a prevalence of 45.28% of cases, as the fifth branch in 27.99%, or as the third branch in 13.39% of cases [19]. In just 5.65% of cases did the OA start at the same level as the FA, and in 0.9% it originated at the level of the LA [19]. In 1.15% the OA was the second branch of the ECA [19]. We consider that for surgeons a bone landmark is more important in identifying an artery than identifying and counting the sequence of ECA branches. Moreover, rare anatomical variants, such as the pentafurcation of the ECA [6], could not respect a certain order of the arterial origins.

Hayashi et al. (2005) studied branching patterns of the ECA just in the carotid triangle, in 49 cadavers [30]. The authors found the origin of the OA on the posterior side of the ECA above the level of the origin of the FA in 57% of specimens, between the origins of the FA and LA in 32%. and below the origin of the LA in 11% [30]. According to these descriptions, it appears that the gonial and supragonial OA origin levels from the ECA were not found, or observed [30]. As Touré (2010) discussed, a supradigastric origin of OA can make it difficult for surgical identification [28]. We found supradigastric types 4 (gonial type) and five (supragonial type) in 28.33% and 23.33%, respectively; this results in an incidence of more than 50% of cases in which the OA originates superior to the posterior belly of the digastric muscle. This is an important incidence to consider when designing flaps with the OA as pedicle.

In almost half of the cases we found here infradigastric types of OA: 1 (infrahyoid, 1.11%); 2 (hyoid, 5.56%); and 3 (infragonial, 40.56%). The infrahyoid type 1 of OA origin is thus seemingly rare. Moreover, the OA and superior thyroid artery origins from the ECA at the same level were not, to our knowledge, previously reported. The tip of the greater horn of the hyoid bone is regarded as a landmark for the localization of the carotid bifurcation, the superior thyroid artery, the LA, and the hypoglossal and superior laryngeal nerves [31]. Surgeons should, however, be aware of hyoid and infrahyoid types of the OA that occasionally add this artery to the list of structures closely related to the greater horn of the hyoid bone.

Different authors previously reported cases of OAs emerging from the ICAs. Newton and Young (1868) were the first who demonstrated by angiography the possibility of the origin of the OA from ICA [14]. Newton and Young found the origin of the OA at 2 cm distally to the carotid bifurcation [32], similarly to Adachi (1928) [32]. Sundick et al. (2014) reported for the first time a three-dimensional reconstruction of an OA originating from the ICA, which correlated with intraoperative images [33]. The aberrant vessel originated from the ICA 1 cm distally to the carotid bifurcation [33]. Benson and Hamer documented in 1988 a series of early reports of abnormal origin of OA [34]. These authors documented that Hyrtle reported in 1841 a case in which the OA and AAP originated in the common trunk from the ICA [35]; such an occipitoauricular trunk was later reported also by Quain [36]. Seidel, quoted by Benson and Hamer, demonstrated by angiography in 1965 all branches of the ACE leaving from the ICA [34,37]. Iwai et al. (2012), as well as Yoshikawa et al. (2013), Uchino et al. (2015), and Özgür et al. (2017), also reported cases with the ICA origin of the OA [15,38,39,40]. Hachem et al. (2004) reported a case with asymptomatic occlusion of the ICA due to the presence of an OA that left the ICA above the carotid sinus and communicated with the vertebral artery on that side [41]. Uchino and Saito (2020) reported also a three cases series, identified by MRI, with OA’s origin from the ICA at the level of the vertebral body of axis [21].

By the present study were found OAs originating in 1.1% from the ICA. According to Lippert and Pabst, the OA originates from the ICA in <0.1% [42]. Uchino et al. (2011) retrospectively documented the MRI files of 2866 patients [43]. They found in 0.21% the ICA origin of OA, usually on the right side [43]. Later, the origin of the OA was documented in a batch of 265 cases [44]. The authors determined the incidence of OA origin from ICA to be 0.2% [44]. Later, Small et al. (2014) used a retrospective cohort of 2602 cases to document arterial origins from the cervical portion of the ICA [45]. These authors found OAs originating from the ICA in 0.49% of cases [45]. The distance between the aberrant OA origin and the carotid bifurcation was variable, ranging from 3 mm to 45 mm [45].

The OA is an important donor artery for posterior cranial fossa revascularization due to its size, anatomic proximity to the recipient vessels and flow [18,22,23,46,47]. It may also be used for bypasses to the anterior cerebral circulation [48]. The OA may also be of use for superselective intraarterial chemotherapy if other routes are unavailable [44]. Therefore, an anatomic mapping of the OA appears mandatory in such cases. When the OA originates from the ICA and CT angiography is not done preoperatively, the catheter inserted into the ICA could cause cerebral infarction [39]. The OA represents the superior pedicle of the trapezius muscle [49]. Thus, the OA is important for the design of the superior trapezian flap. Localization and preservation of the OA in the upper cervical region is essential when aiming to achieve a myocutaneous trapezian flap for reconstruction [1].

## 5. Conclusions

The origin of the OA is variable in different subjects, in most cases being symmetrical or relatively symmetrical. For surgical planning or prior to endovascular approaches, the topography of the OA origin should be carefully documented as it may be located from an infrahyoid to a supragonial level.

## Figures and Tables

**Table 1:** Variability of the vertical level of origin of the occipital artery (OA). ICA: internal carotid artery.

Vertical Levels of Origin of the OA (N = 180)	Count, %
type 1—infrahyoid level of origin	2 (1.11%)
type 2—hyoid level of origin	10 (5.56%)
type 3—suprahyoid level of origin	73 (40.56%)
type 4—gonial level of origin	51 (28.33%)
type 5—supragonial level of origin	42 (23.33%)
type 6—ICA origin	2 (1.11%)

**Table 2:** Prevalence of the types of vertical origin of the occipital artery, on sides, in the general lot, males, and females.

Type	General Lot (90 Cases)		Males (53 Cases)		Females (37 Cases)	
	Right Side	Left Side	Right Side	Left Side	Right Side	Left Side
1	2.22%	0	0	0	5.41%	0
2	3.33%	7.78%	3.77%	7.55%	2.7%	8.11%
3	36.67%	44.44%	39.62%	49.06%	32.43%	37.84%
4	25.56%	31.11%	22.64%	26.42%	29.73%	37.84%
5	30%	16.67%	30.19%	16.98%	29.73%	16.22%
6	2.22%	0	3.77%	0	0	0

## Data Availability

No new data were created or analyzed in this study. Data sharing is not applicable to this article.
